# Connective tissue mast cells store and release noradrenaline

**DOI:** 10.1186/s12576-023-00883-3

**Published:** 2023-10-12

**Authors:** Yusuke Otani, Soichiro Yoshikawa, Kei Nagao, Takehiro Tanaka, Shinichi Toyooka, Atsushi Fujimura

**Affiliations:** 1 Department of General Thoracic Surgery and Breast and Endocrinological Surgery, Okayama University Graduate School of Medicine, Dentistry and Pharmaceutical Sciences, 2-5-1 Shikata-cho, Kita-ku, Okayama, 700-8558 Japan; 2 Department of Cellular Physiology, Okayama University Graduate School of Medicine, Dentistry and Pharmaceutical Sciences, 2-5-1 Shikata-Cho, Kita-Ku, Okayama, 700-8558 Japan; 3 Department of Pathology and Oncology, Okayama University Graduate School of Medicine, Dentistry and Pharmaceutical Sciences, 2-5-1 Shikata-cho, Kita-ku, Okayama, 700-8558 Japan; 4 Neutron Therapy Research Center, Okayama University, 2-5-1 Shikata-cho, Kita-ku, Okayama, 700-8558 Japan

**Keywords:** Mast cells, Connective tissue mast cells, Noradrenaline, Immunoelectron microscopy, SLC22A3

## Abstract

Mast cells are present in mucosal and connective tissues throughout the body. They synthesize and release a wide variety of bioactive molecules, such as histamine, proteases, and cytokines. In this study, we found that a population of connective tissue mast cells (CTMCs) stores and releases noradrenaline, originating from sympathetic nerves. Noradrenaline-storing cells, not neuronal fibers, were predominantly identified in the connective tissues of the skin, mammary gland, gastrointestinal tract, bronchus, thymus, and pancreas in wild-type mice but were absent in mast cell–deficient *W-sash c-kit* mutant *Kit*^*W−sh/W−sh*^ mice. In vitro studies using bone marrow–derived mast cells revealed that extracellular noradrenaline was taken up but not synthesized. Upon ionomycin stimulation, noradrenaline was released. Electron microscopy analyses further suggested that noradrenaline is stored in and released from the secretory granules of mast cells. Finally, we found that noradrenaline-storing CTMCs express organic cation transporter 3 (Oct3), which is also known as an extraneuronal monoamine transporter, SLC22A3. Our findings indicate that mast cells may play a role in regulating noradrenaline concentration by storing and releasing it in somatic tissues.

**Supplementary Information:**

The online version contains supplementary material available at 10.1186/s12576-023-00883-3.

## Background

The control of organ and tissue function by the sympathetic nervous system is dynamic, yet very tightly controlled [1–3]. The neurotransmitter noradrenaline must be properly released and retrieved for sympathetic nerves to act on target organs. Although the molecular mechanisms by which noradrenaline release, uptake, and metabolism are managed are well understood [4–6], it remains unclear how noradrenaline released from the sympathetic nervous system synchronously regulates the physiology of target organs. For example, textbooks describing “sympathetic control of target organs” frequently feature illustrations in which sympathetic nerves interface directly with target organs via synapses. However, it remains unknown how and why cells without synapses are affected by noradrenaline. Moreover, to achieve a synchronous response, noradrenaline must be available to most cells in the tissue. However, noradrenaline supply by diffusion from the area of sympathetic innervation would be insufficient for synchronous responses. To grasp an understanding for these questions, here, we initially aimed to determine the spatial distribution of noradrenaline in various tissues.

For this purpose, immunostaining was performed using antibodies against noradrenaline in various somatic tissues. We initially expected a gradient of noradrenaline concentrations within the organs. Contrary to our expectations, we found that a population of noradrenaline-containing cells was scattered mainly in the connective tissues of each organ. From histological and immunostaining results, we identified these cells as connective tissue mast cells (CTMCs).

Mast cells synthesize or take up and store a variety of bioactive substances such as histamine and serotonin and play a central role in allergic reactions, mainly in vivo [7, 8]. The chemical mediators taken up by mast cells have been widely studied. For example, in the 1960s and 1970s, mast cells were reported to take up, store, and subsequently release histamine and serotonin [9–11]. However, to the best of our knowledge, there are no studies that discuss the possibility of mast cells storing and releasing noradrenaline, and only one study has confirmed the effect of noradrenaline on histamine release by mast cells [12]. Intriguingly, mast cells were shown to be involved in the regulation of heart rate in response to anaphylaxis in mice [13] and in the modulation of noradrenaline release from cardiac sympathetic nerve terminals [14]. Similarly, macrophages in adipose tissue were shown to take up noradrenaline released from sympathetic nerve terminals and modulate sympathetic nerve function [15]. Regarding these past studies, we here aimed to examine the possibility that mast cells might take up, store, and release noradrenaline. Our study would be physiologically and pathophysiologically important from the viewpoint of novel function of mast cells as a spatial distributer of noradrenaline.

## Materials and methods

### Animals

**Fig. 1:**
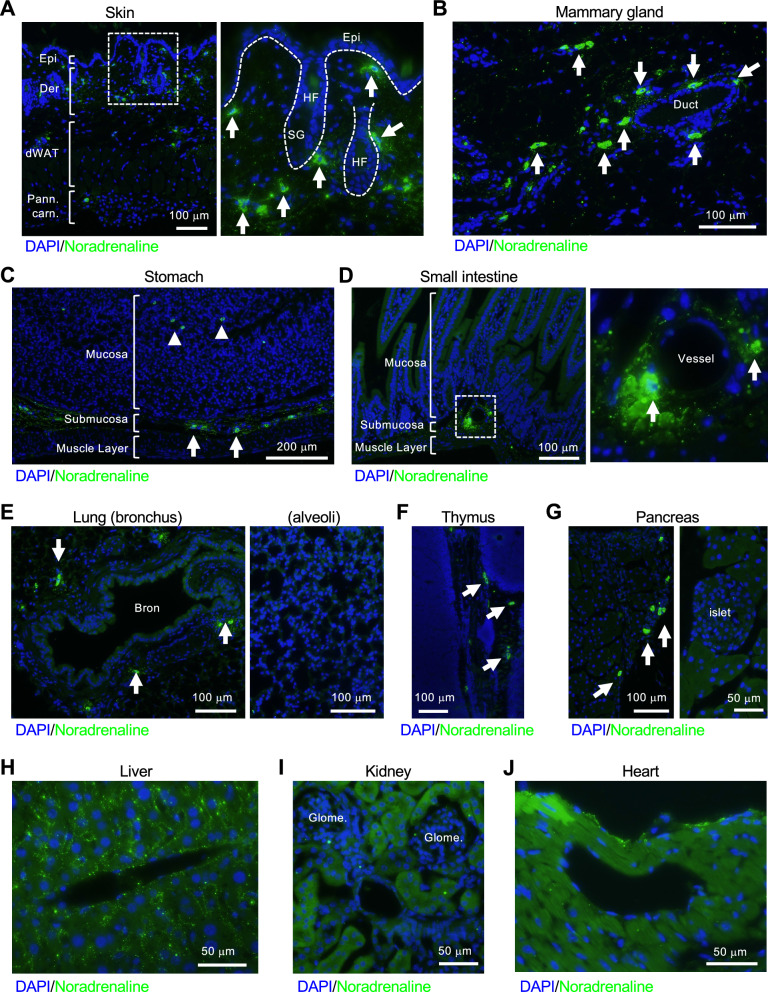
Discovery of noradrenaline-storing cells in mouse tissues. **A** Noradrenaline-storing cells were observed from the dermis to hypodermis in the skin of mice. They were abundant in the epidermis and around hair follicles (arrows). Scale bars: 100 µm. *Epi* epidermis, *Der* dermis, *dWAT* dermal white adipose tissue, *Pann. carn.* panniculus carnosus, *HF* hair follicle, *SG*: sebaceous gland. **B** Noradrenaline-storing cells were observed in the periductal area (arrows) and in adipose tissues in mammary glands. Scale bar: 100 µm. Duct: mammary duct. **C** and **D** Noradrenaline-storing cells were abundant in the submucosa (arrows), and few were observed in the mucosa (arrowheads) in stomach (**C**) and small intestine (**D**). Scale bars: 200 µm in (**C**) and 100 µm in (**D**). **E** Noradrenaline-storing cells were abundant in the peribronchial area (arrows) of the lung but not in the alveoli. Scale bars: 100 µm. Bron: bronchus. **F** Noradrenaline-storing cells were found in the interstitium of the thymus but were not present in the parenchyma (arrows). Scale bar: 100 µm. **G** Noradrenaline-storing cells were absent in the islets and acini of the pancreas but present in the surrounding stroma (arrows). Scale bars: 100 or 50 µm. **H**–**J** Cells were not found in the liver (**H**), kidney (**I**), and heart (**J**). Scale bars: 50 µm. *Glome.* glomerulus. Three independent experiments were performed in each organ, and the representative pictures are shown in this Figure. Representative images of negative controls for primary antibody are shown in Additional file 1

**Fig. 2:**
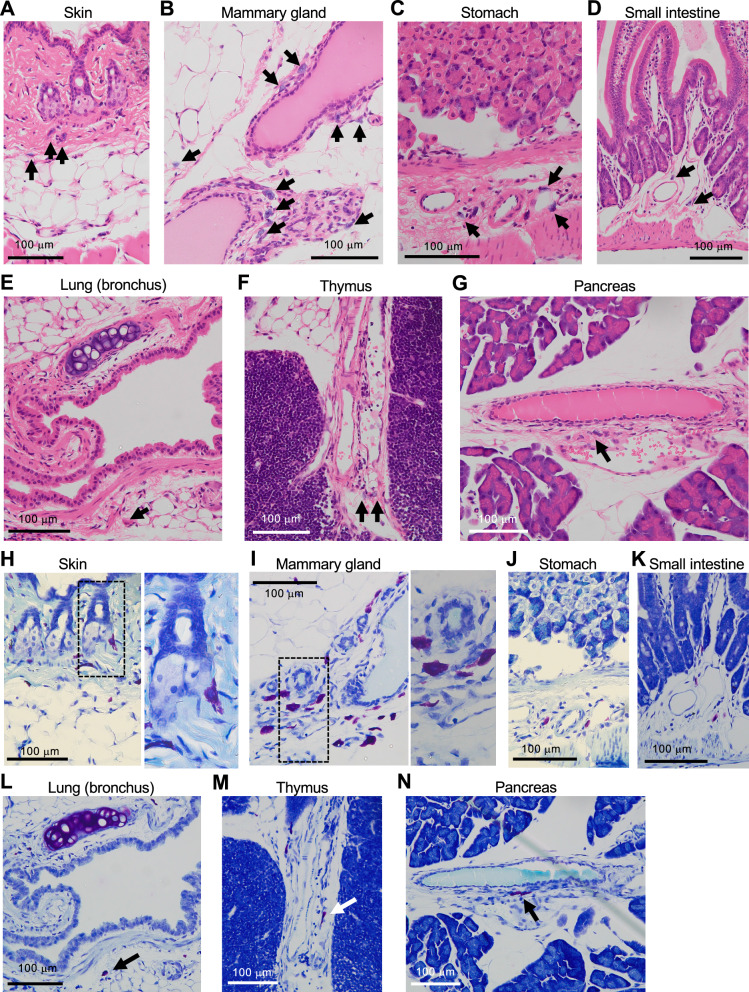
HE and toluidine-blue staining indicate that noradrenaline-storing cells are mast cells. **A**–**G** In the areas of each tissue where noradrenaline-storing cells are observed in immunofluorescent analyses shown in Fig. 1, cells with storage granules were stained with basophilic color (arrows). The morphology and localization of stained cells indicates that they were mast cells. Scale bars: 100 µm. **H**–**N** Toluidine blue staining was performed to confirm the presence of mast cells in the same areas. Scale bars: 100 µm

**Fig. 3:**
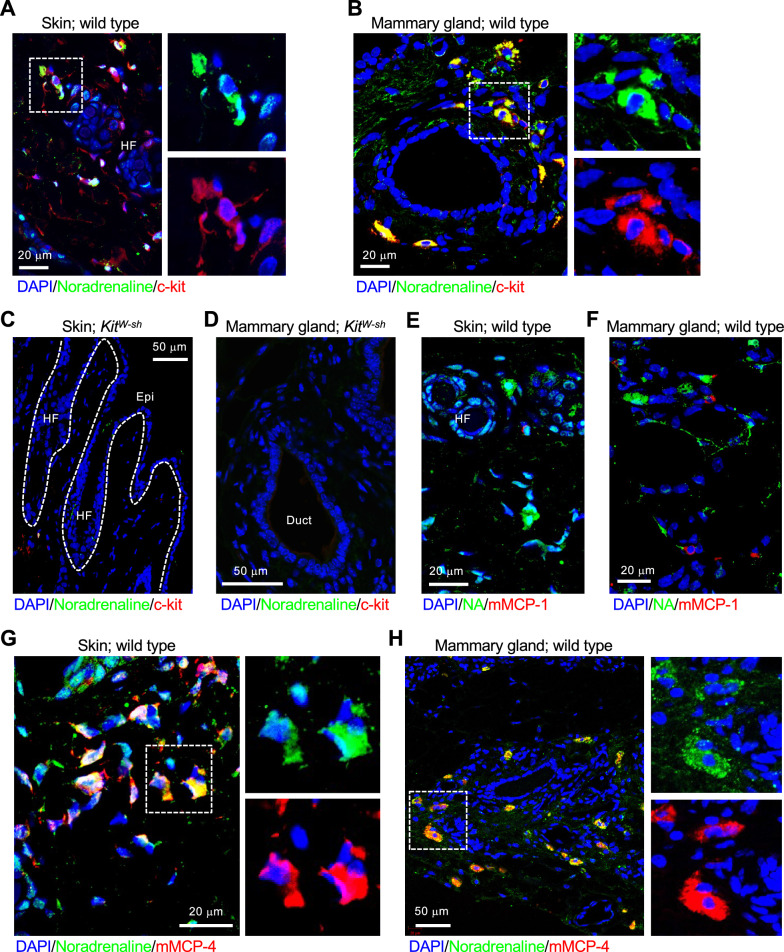
Connective tissue mast cells store noradrenaline. **A** and **B** Representative images of immunofluorescent staining in mouse skin (**A**) and mammary gland (**B**) using antibodies against noradrenaline (green) and c-kit (red), a marker for mast cells. Noradrenaline-storing cells are positive for c-kit. Scale bars: 20 µm. HF: hair follicle. **C** and **D** In the skin (**C**) and mammary gland (**D**) of mast cell–deficit mice, no noradrenaline and c-kit signals were observed, confirming that noradrenaline-storing cells in the tissues are mast cells. Scale bars: 50 µm. *HF* hair follicle, *Epi* epidermis, *Duct* mammary duct. **E** and **F** Representative images of immunofluorescent staining in normal mouse skin (**E**) and mammary gland (**F**) using antibodies against noradrenaline (green) and mMCP-1 (red), a marker for mucosal mast cells (MMC). Scale bars: 20 µm. HF: hair follicle. **G** and **H** Noradrenaline-storing cells are positive for mMCP-4, a marker for connective tissue mast cells (CTMCs), confirming that the cells are CTMCs. Scale bars: 20 or 50 µm. Three independent experiments were performed in each organ, and the representative pictures are shown in this Figure

**Fig. 4:**
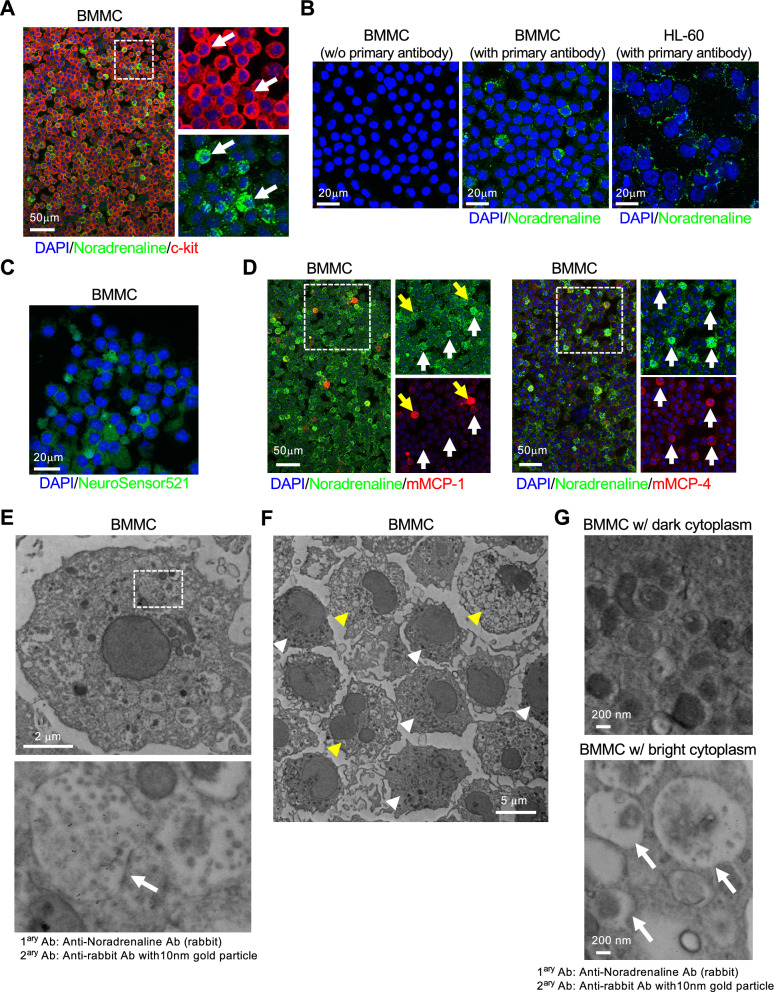
In vitro studies using bone marrow-derived mast cells confirm that mast cells store noradrenaline. **A** Representative images of bone marrow-derived mast cells (BMMCs) immunostained with antibodies against noradrenaline and c-kit, suggesting that the mast cells store noradrenaline. Note the heterogeneous staining pattern of noradrenaline (arrows). Scale bar: 50 µm. Four independent experiments were performed, and a representative image is shown. **B** BMMCs or HL-60 cells are stained with antibodies against noradrenaline. The negative control panel confirmed that signals were from the primary antibodies. Note that no signal was observed in HL-60 cells. Scale bars: 20 µm. Three independent experiments were performed, and the representative images are shown. **C** Representative image of BMMCs treated with NeuroSensor 521, a chemical fluorescent probe for noradrenaline, confirming that the mast cells store noradrenaline. Scale bar: 20 µm. Three independent experiments were performed, and a representative image is shown. **D** Representative image of BMMCs stained with antibodies against noradrenaline and mMCP-1 (left panel) or mMCP-4 (right panel). Noradrenaline-storing mast cells are negative for mMCP-1 but positive for mMCP-4 (yellow and white arrows), confirming that the noradrenaline-storing mast cells are CTMCs. Scale bars: 50 µm. Three independent sets of experiment were performed, and the representative images are shown. **E** Representative image of immunoelectron microscopic analysis of BMMCs using noradrenaline antibodies and secondary antibodies labeled with 10 nm gold nanoparticles. Note that the particles were abundantly detected in the secretory granule (arrow). Scale bar: 2 µm. Two independent experiments were performed, and a representative image is shown. **F** and **G** Gold nanoparticles indicate that noradrenaline was predominantly found in cells with bright cytoplasm (arrows). Scale bars: 5 µm or200 nm

**Fig. 5:**
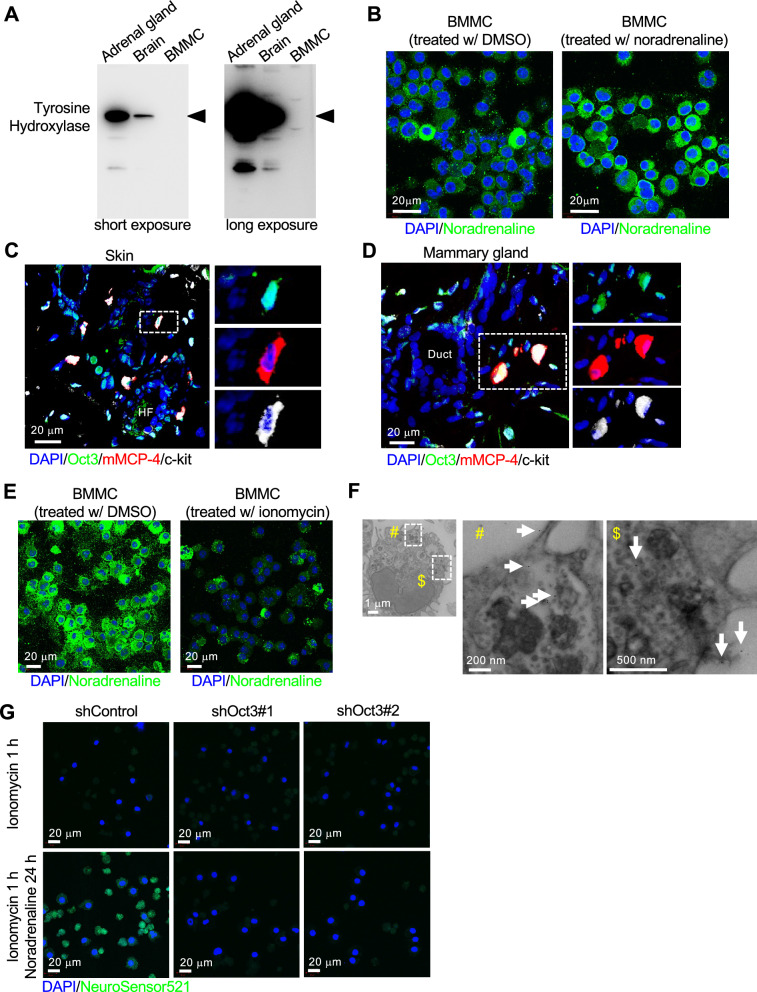
Mast cells store and release noradrenaline. **A** Western blotting images of tyrosine hydroxylase (TH) indicate that BMMCs do not express TH, suggesting that BMMCs could not synthesize noradrenaline. **B** Immunofluorescent images using noradrenaline antibodies in BMMCs treated with DMSO (left) or 10 nM noradrenaline (right) for 24 h. After noradrenaline treatment, the signal was much stronger than that of negative control, suggesting that BMMCs could take up extracellular noradrenaline into the cytoplasm. Scale bars: 20 µm. Four independent experiments were performed, and the representative images are shown. **C** and **D** Representative images of immunofluorescent staining in mouse skin (**C**) and mammary gland (**D**) using antibodies against Oct3 (green), mMCP-4 (red), and c-kit (white). Note that noradrenaline-storing CTMCs are positive for Oct3. Scale bars: 20 µm. *HF* hair follicle, *Duct* mammary duct. Three independent experiments were performed in each organ, and the representative pictures are shown in this Figure. Representative images of negative controls for primary antibodies are shown in Additional file 1. **E** Stimulation of noradrenaline-storing BMMCs with ionomycin indicates that BMMCs could release noradrenaline. Scale bars: 20 µm. Four independent experiments were performed, and the representative images are shown. **F** Representative image of immunoelectron microscopic analysis of ionomycin-stimulated BMMCs using noradrenaline antibodies and secondary antibodies labeled with 10 nm gold nanoparticles. Note that the particles are found in the secreting granule (arrow). Scale bars: 1 µm, 200 nm, or 500 nm. Two independent experiments were performed, and a representative image is shown. **G** Representative images of shRNA-induced BMMCs treated with NeuroSensor 521, confirming that Oct3 is required to take up noradrenaline. Scale bars: 20 µm. Two independent sets of experiment were performed, and the representative images are shown

All animal experiments were performed with permission from and in accordance with all guidelines put forth by the committees of Okayama University (approval number: OKU-2022436 and OKU-2022474 for animal experiments and 21134 for recombinant DNA experiments for *W-sash c-kit* mutant *Kit*^*W−sh/W−sh*^ mice). All histological experiments were performed on female 10-week-old mice. Three C57BL/6 mice were obtained from Charles River (Yokohama, Japan) and used as “wild type” for histological analyses shown in Figs. 1, 2, 3, and 5. *W-sash c-kit* mutant *Kit*^*W−sh/W−sh*^ mice were kindly provided by Dr. Katsuko Sudo (Tokyo Medical University) and three *Kit*^*W−sh/W−sh*^ mice were used for histological analyses shown in Fig. 3. For each isolation of bone marrow-derived mast cells (BMMCs), three female 7-week-old C57BL/6 mice were used, and four independent isolations of BMMCs were performed for Figs. 4 and 5. In all animal experiments, mice were euthanized by cervical dislocation.

### Immunofluorescent analysis

Immunofluorescence analyses of paraffin-embedded sections were performed as previously described [16] with minor modifications. Sections for the analyses were prepared to a thickness of 4 µm on APS-coated slide glasses (Matsunami glass, Osaka, Japan) and deparaffinized in a serial incubation in xylene (5 min, twice), 95% ethanol (5 min), 90% ethanol (5 min), 70% ethanol (5 min) and, phosphate-buffered saline (PBS) at room temperature (20 to 25 °C). Deparaffinized sections were blocked with 1% Block Ace reagent (KAC, Kyoto, Japan) for 1 h at room temperature, followed by overnight primary antibody incubation at 4 °C. After washing with PBS three times, the sections were incubated with the secondary antibody for 1 h at room temperature in a humid and dark chamber. The stained sections were then mounted with DAPI (SouthernBiotech, Birmingham, USA). Three sections of each organ obtained from different mice were stained and the representative pictures were shown in Fig. 1. For immunofluorescence analyses of cells, we followed a previously described method [17] with minor modifications. Briefly, prior to staining, BMMCs were attached onto glass slides using Cytospin (Thermo Fisher Scientific, Waltham, USA), a cytocentrifuge machine. After fixing with 4% paraformaldehyde (PFA) for 30 min at room temperature and washing briefly, the cells were blocked with 1% Block Ace reagent for 1 h at room temperature, followed by overnight primary antibody incubation at 4 °C. Secondary antibody incubation and mounting were performed as described above. Staining with NeuroSensor 521 (Sigma-Aldrich, St. Louis, USA) was performed as described in previous literature [18]. Imaging was performed using a confocal laser-scanning microscope LSM780 (Carl Zeiss, Oberkochen, Germany). The following are the antibodies used in this study, listed as [Antigen/Source/Identifier/Dilution]: [Noradrenaline/Abcam/ab8887/1:200]; [c-Kit/BioLegend/105802/1:200]; [mMCP-1/Thermo Fisher Scientific/14–5503-82/1:100]; [mMCP-4/Abcam/ab92368/1:200]; [SLC22A3/Alomone labs/ACT-013/1:200]; [Donkey anti-rabbit IgG Alexa Fluor Plus 488/Thermo Fisher Scientific/A32790/1:500]; [Donkey anti-rat IgG Alexa Fluor 594/Thermo Fisher Scientific/A21209/1:500]; [Donkey anti-rat IgG Alexa Fluor 647/Thermo Fisher Scientific/A78947/1:500]; [Donkey anti-goat IgG Alexa Fluor 594/Thermo Fisher Scientific/A32758/1:500].

### HE and toluidine-blue staining

HE staining was performed as previously reported [19]. Sections were prepared to a thickness of 4 µm and stained using Tissue-Tek Prisma Plus, an automated slide stainer (Sakura Finetek, Tokyo, Japan). Gill’s hematoxylin V (Muto Pure Chemical, Tokyo, Japan) and eosin-Y (Sigma-Aldrich) were used. Toluidine-blue staining was performed manually using the following protocol. Briefly, after deparaffinization, the sections were stained with 0.05% toluidine-blue (pH 4.1, Muto Pure Chemical) for 30 min at room temperature, followed by brief washing and mounting. Imaging was performed using a Nikon Eclipse 80i optical microscope (Nikon, Tokyo, Japan) and cellSense Standard software (Olympus, Tokyo, Japan).

### Western blotting

Western blotting was performed as previously described [20]. Briefly, after lysis using lysis buffer (20 mM Tris–HCl (pH 7.5), 150 mM NaCl, 1 mM ethylenediaminetetraacetic acid, 1 mM Ethylene glycol-bis(2-aminoethylether)-N,N,N',N'-tetraacetic acid, 1% Triton X-100, cOmplete protease inhibitor cocktail (Roche, Basel, Switzerland), PhosSTOP phosphatase inhibitor cocktail (Roche)) and boiling in sodium dodecyl sulfate (SDS) sample buffer (50 mM Tris–HCl (pH 6.5), 100 mM dithiothreitol, 2% SDS, 1.5 mM bromophenol blue, 1.075 M glycerol), the samples were subjected to SDS–polyacrylamide gel electrophoresis (SDS-PAGE) and transferred to a polyvinylidene difluoride membrane (0.45 µm pore size, Millipore, Burlington, MA, USA). The membranes were then subjected to immunodetection using antibodies against tyrosine hydroxylase (Novus Biologicals, Centennial, USA). The signals were detected using a ChemiDoc Touch imaging system (Bio-Rad, Hercules, USA). The following are the antibodies used in this study, listed as [Antigen/Source/Identifier/Dilution]: [Tyrosine hydroxylase/Novus Biologicals /NB300-109/1:2000]; [c-Kit/BioLegend/105802/1:200]; [Goat anti-rabbit IgG HRP-linked/Cell Signaling Technology/7074S/1:2000].

### Cell culture

Mouse bone marrow-derived mast cells (BMMCs) were isolated as previously described [21]. Briefly, BMMCs were prepared by culturing bone marrow cells in the presence of recombinant mouse SCF (15 ng/mL. BioLegend) and mouse IL-3 (3 ng/mL, BioLegend) for 4 weeks. Human promyelocytic leukemia cells HL-60 were cultured in RPMI-1640 medium (Fujifilm Wako Pure Chemical, Osaka, Japan) supplemented with 20% fetal bovine serum (Nichirei Biosciences, Tokyo, Japan) and antibiotics (Fujifilm Wako Pure Chemical). Lentivirus preparation and infection were performed as previously reported [22]. Briefly, 293FT cells were transfected with 10 µg of pLKO.1 puro lentiviral backbone plasmid (Addgene #8453), 7.5 µg of psPAX2 (Addgene #12260), and 2.5 µg of pMD2.G (Addgene #12259) using TransIT-LT1 transfection reagent (TaKaRa Bio) and Opti-MEM (Thermo Fisher Scientific). After 24 h of transfection, the medium was changed to 10 mL of BMMC medium, and the cells were cultured for 48 h. Virus-containing medium was harvested and filtered with polysulfone membrane (0.45 µm pore size, Kurabo, Osaka, Japan). The following are the target sequences of shRNA used in this study:Murine Slc22a3 #1 [CAGGCTCATCATTTACTTAAT]; Murine Slc22a3 #2 [CGCTCATCCTTATGTTTGCTT]; Control [CCTAAGGTTAAGTCGCCCTCG]. The oligonucleotides containing the target sequence and hairpin loop were synthesized and obtained from FASMAC (Atsugi, Japan) and cloned into AgeI/EcoRI-digested pLKO.1 puro lentiviral vector by ligating with DNA ligation kit (TaKaRa Bio).

### Electron microscopy

Immunoelectron microscopy was performed as previously reported [23]. Cells were fixed with 4% PFA and 0.1% glutaraldehyde for 1 h at room temperature, followed by fixation with 1% osmium tetroxide and 1.5% potassium ferrocyanide for 30 min at room temperature. The samples were then mounted in LR-White Hard resin (Okenshoji, Tokyo, Japan) and cut at 80 nm thickness. The sections were blocked with 1% bovine serum albumin and 10% goat serum, followed by overnight primary antibody incubation with anti-noradrenaline antibodies (Abcam, Cambridge, UK, 1:50) at 4 °C. After washing three times with PBS, the sections were treated with goat anti-rabbit IgG conjugated with 10 nm gold nanoparticles (BBI solutions, Gwent Crumlin, UK, 1:100) for 1 h at room temperature, followed by further treatment with uranyl acetate. Imaging was performed using transmission electron microscope (Hitachi H-7650) (Hitachi High-Tech, Tokyo, Japan).

## Results

### Noradrenaline-storing cells are present in connective tissues throughout the body

Using immunofluorescent analysis, we identified a population of cells stained with anti-noradrenaline antibodies in mouse tissues, including the skin, mammary gland, stomach, small intestine, lung, thymus, and pancreas. In the skin, these cells were scattered widely from the dermis to the hypodermis and were especially abundant in the vicinity of the epidermis and around hair follicles (Fig. 1A). In mammary tissues, most of these cells were present in the periductal area, and a few were present in adipose tissues (Fig. 1B). In the stomach and small intestine, these cells were mostly present in the submucosa, and a few were present in the mucosa but not in the muscularis (Fig. 1C, D). In the lungs, these cells were found exclusively in the peribronchial area and not in the alveoli (Fig. 1E). In the thymus, these cells were not present in the parenchyma but were scattered in the interstitium (Fig. 1F). In the pancreas, these cells were absent in the islets and acini but were scattered in the surrounding stroma (Fig. 1G). Unlike the above tissues, in the liver, the kidney, and the heart, noradrenaline staining was observed diffusely in hepatocytes, renal tubular epithelial cells, and cardiocytes, respectively (Fig. 1H–J). These results indicate that noradrenaline-storing cells are abundant in the connective tissues of certain organs.

### Hematoxylin–eosin and toluidine-blue staining indicate that noradrenaline-storing cells are mast cells

Next, we evaluated hematoxylin–eosin (HE)-stained images of the areas of the serial sections in which these cells were identified abundantly to determine the characteristics of noradrenaline-storing cells. As shown in Fig. 2, we found several cells that store granules stained with basophilic color in the dermis and hypodermis of the skin (Fig. 2A), the periductal area and adipose tissues of the mammary gland (Fig. 2B), the submucosa of the stomach and small intestine (Fig. 2C, D), the peribronchial area of the lung (Fig. 2E), the interstitium of the thymus (Fig. 2F), and the interstitium of the pancreas (Fig. 2G). Cell morphology and localization suggested that they were mast cells. To confirm this, we further stained the serial sections of the tissues with toluidine blue, because mast cells are known to exhibit metachromasia, causing them to appear reddish-purple after staining. When sections of organs in which noradrenaline-stained cells were localized were stained with toluidine blue, cells showing metachromasia were observed in areas where noradrenaline-stained cells were abundant (Fig. 2H–N). These data suggest that noradrenaline-storing cells are mast cells.

### Noradrenaline-storing mast cells are connective tissue mast cells

To verify that the noradrenaline-storing cells observed in the connective tissue of each organ were mast cells, we performed immunostaining of mouse skin and mammary tissue using antibodies against noradrenaline and c-kit, a marker of mast cells. In both tissues, a large number of cells with strong signals was observed in the connective tissue of the organs (Fig. 3A, B). Furthermore, similar analysis of skin and mammary tissue from *Kit*^*W−sh*^ “sash” mice lacking mast cells showed no c-kit-positive cells and no noradrenaline-positive cells (Fig. 3C, D). These data clearly indicated that the identified noradrenaline-storing cells were mast cells.

Mast cells are classified into several subclasses according to the content of their secretory granules. In humans, mast cells are classified as MC_T_ (tryptase^+^) and MCTC (tryptase^+^/chymase^+^/mast cell-carboxypeptidase^+^) based on the protease they contain [24]. In mice, mast cells are classified into mucosal mast cells (MMCs) and CTMCs based on their localization, and the protease they express: MMCs express mouse mast cell protease (mMCP)-1, and CTMCs express mMCP-4 [25]. To determine the type of noradrenaline-storing mast cells, we immunostained mouse skin and mammary glands with antibodies against noradrenaline, mMCP-1 or mMCP-4. We found that there was no overlapping signal for noradrenaline and mMCP-1 (Fig. 3E, F), but there were many overlapping signals for noradrenaline and mMCP-4 (Fig. 3G, H). These data indicated that noradrenaline-storing mast cells are CTMCs.

### Mast cells store and release noradrenaline

Next, we aimed to verify how CTMCs harbor noradrenaline in their cells. To this end, we conducted in vitro validation using primary cultured mast cells derived from the bone marrow of wild-type mice. Mast cells mature in peripheral tissues but not in lymphoid organs [26]. The differentiation process begins with the release of mast cell progenitors derived from the granulocyte/monocyte progenitor cell population from the bone marrow, followed by their maturation in peripheral tissues [27, 28]. A method for inducing mast cells in vitro using bone marrow-derived mast cell progenitors has been established. Mast cells are obtained by culturing a cell population derived from bone marrow in a medium containing stem cell factor (SCF) and interleukin-3 (IL-3) for approximately 1 month [29, 30]. Following this protocol, with some modifications, a population of bone marrow cells derived from mice was collected and cultured in medium containing 15 ng/mL and 3 ng/mL of SCF and IL-3, respectively, for 1 month. Cells were attached to glass slides using a cytospin system (cytocentrifugation) and subsequently immunostained with antibodies against noradrenaline and c-kit. Most cells expressed c-kit, suggesting that we succeeded in obtaining bone marrow-derived mast cells (BMMCs) (Fig. 4A). Interestingly, not all BMMCs had strong noradrenaline signals, and heterogeneous staining was observed (Fig. 4A, arrows indicate strong noradrenaline signals). The specificity of staining by noradrenaline antibodies in mast cells was confirmed by results obtained for negative controls (without primary antibodies) and other hematopoietic cell cultures (HL-60 cells; human promyelocytic leukemia cells) (Fig. 4B). To further confirm the presence of noradrenaline in BMMCs, we stained the fixed BMMCs with NeuroSensor 521, a chemical probe that fluoresces upon chemical binding to noradrenaline [18] and observed heterogeneity staining, similar to immunostaining using noradrenaline antibodies (Fig. 4C). Similar to the results of immunostaining in mouse skin and mammary tissue, most noradrenaline-positive BMMCs were positive for mMCP-4, suggesting that they were CTMCs (Fig. 4D). Furthermore, immunoelectron microscopic analysis of BMMCs using noradrenaline antibodies and secondary antibodies labeled with 10 nm gold particles indicated the presence of noradrenaline within the secretory granules of BMMCs (Fig. 4E, arrow). Similar to the results of immunostaining and analysis using NeuroSensor 521, cellular heterogeneity was observed in the noradrenaline signal in immunoelectron microscopy (Fig. 4F). Fewer noradrenaline signals were observed in cells with dark cytoplasm (white arrowheads in Fig. 4F), whereas more signals were observed in cells with light cytoplasm (yellow arrowheads in Fig. 4F) (Fig. 4G: white arrows indicate noradrenaline-containing granules). These data indicate that BMMCs store noradrenaline in secretory granules.

Next, we examined the origin of the noradrenaline stored in the secretory granules of mast cells. We first examined the possibility that mast cells themselves synthesize noradrenaline through a biosynthetic pathway that begins with L-tyrosine, which is converted to L-dopa by tyrosine hydroxylase (TH). L-dopa is converted to dopamine by dopa decarboxylase (DDC), and dopamine is converted to noradrenaline by dopamine beta-hydroxylase (DbH) [31]. TH is the rate-determining factor in this pathway. Western blot analysis of BMMCs showed that the BMMC did not express TH, whereas the positive control adrenal and cerebral tissues showed strong expression levels of TH (Fig. 5A). Next, we tested the possibility that mast cells take up extracellular noradrenaline into the cells. BMMCs cultured in a medium containing dimethyl sulfoxide (DMSO) or 10 nM noradrenaline for 24 h were immunostained with an antibody against noradrenaline. Noradrenaline-treated BMMCs showed stronger noradrenaline signals than DMSO-treated BMMCs did (Fig. 5B). A previous report showed that noradrenaline is taken up by cells via organic cation transporter 3 (Oct3) [32]. Therefore, we examined whether Oct3 is expressed in mast cells in mouse skin and mammary glands by immunostaining and found that c-kit-positive/mMCP4-positive CTMCs expressed Oct3 (Fig. 5C, D). These data suggested that mast cells take up extracellular noradrenaline.

Finally, to determine whether BMMCs can release noradrenaline, we induced mast cell degranulation and performed immunofluorescence analysis. BMMCs cultured in medium containing 10 nM noradrenaline for 24 h were stimulated with DMSO or 10 µM ionomycin, a chemical compound that induces mast cell degranulation [33], for 1 h and immunostained with an antibody against noradrenaline. We found that degranulation stimulation with ionomycin attenuated noradrenaline staining, suggesting that noradrenaline was released from BMMCs (Fig. 5E). Immunoelectron microscopy analysis also indicated that noradrenaline-positive granules were secreted (Fig. 5F). To further confirm that BMMCs take up extracellular noradrenaline, we infected BMMCs with lentiviruses expressing shRNA against Oct3. After 72 h, the cells were stimulated with 10 µM ionomycin for 1 h, cultured in medium containing 10 nM noradrenaline for 24 h, and stained with NeuroSensor 521. Oct3 knockdown significantly attenuated NeuroSensor 521 staining (Fig. 5G). These data suggest that the noradrenaline accumulated in CTMCs is not synthesized de novo but is taken up from the outside via Oct3 and can be released from these cells during secretion and degranulation in a manner similar to other chemical mediators.

## Discussion

In this study, we found noradrenaline-storing cells in the connective tissue areas of mouse organs (Fig. 1). Histological and immunohistological analyses using mast cell-deficient mice confirmed that the identified cells were mast cells (Figs. 2 and 3). Among the mast cell subclasses in mice, the noradrenaline-storing cells were confirmed to be CTMCs (Fig. 3). These data were corroborated by in vitro analysis of BMMCs, and immunoelectron microscopy demonstrated that some secretory granules in BMMCs contained noradrenaline (Fig. 4). Noradrenaline in mast cells is not synthesized de novo in the cells themselves but is taken up from the extracellular environment (Fig. 5). Mast cells are known to release various bioactive chemical mediators such as histamine and serotonin, and our findings suggest that noradrenaline is also released from mast cells upon degranulation stimulation (Fig. 5). In this study, we used ionomycin to induce degranulation. Ionomycin is an ionophore that increases intracellular calcium concentration and is widely used to induce degranulation, which releases liquid factors such as histamine from mast cells [33]. The noradrenaline-storing CTMCs we found in this study secrete noradrenaline extracellularly by ionomycin-induced degranulation as well as histamine secretion (Fig. 5). Degranulation is a regulatory exocytosis triggered in response to external stimuli and is a compound exocytosis with fusion of vesicles during the opening release. In human body, degranulation in mast cells occurs when allergic antigens bind to the complex of FceRI and IgE on mast cells, thereby increasing the intracellular calcium concentration. This suggests that accumulated noradrenaline may be involved in allergic reactions. Interestingly, mast cells have been reported in a mouse model to be essential for the induction of tachycardia in anaphylactic reactions [13]. Since noradrenaline acts to increase heart rate, the accumulation and release of noradrenaline in CTMCs may act to prevent a life-threatening situation caused by anaphylactic reactions. These results indicate that mast cells store noradrenaline as a novel chemical mediator.

Noradrenaline is a neurotransmitter in the sympathetic nervous system; it is released from sympathetic nerves as an agonist, mainly for α1 and β1 adrenergic receptors in target organs and tissues [34]. For example, in smooth muscles that express high levels of α1-adrenergic receptors, activation of phospholipase C (PLC) via G-protein (Gq), which acts downstream of the α1-adrenergic receptor, increases intracellular calcium concentrations via inositol triphosphate and diacylglycerol production [35]. As shown in Figs. 1, 2 and 3, noradrenaline-storing CTMCs are abundant in the vicinity of blood vessels, tracheas, and mammary ducts, and they can release noradrenaline into cells. Based on these facts, noradrenaline-storing CTMCs may fine-tune sympathetic smooth muscle contraction in addition to the canonical role of sympathetic nerves. Alternatively, pooled noradrenaline in CTMCs may augment sympathetic effects by releasing it synchronously with sympathetic nerve activity. To verify these hypotheses, it is necessary to comprehensively evaluate sympathetic nerve activity and physiological responses in mast cell-deficient mice as well as to conduct transplantation experiments using wild-type mouse–derived mast cells in deficient mice, which will be the subject of future work.

Noradrenaline also plays an important role in cancer tissues. For example, it has been reported that elevated noradrenaline levels in response to stress enhance cell migration in prostate cancer [36]. In oral cancer, noradrenaline exacerbates tumor progression via β2-adrenergic receptor stimulation [37]. We observed that noradrenaline-storing mast cells, which we discovered in this study, were also localized in the vicinity of the breast cancer tissues (data not shown). In future, it will be necessary to verify whether these mast cells have any effect on cancer progression. For this purpose, xenografts should be generated using cancer cells with the same genetic background as wild-type and sash mice, and tumor progression should be evaluated.

In addition to noradrenaline, mast cells take up various bioactive substances. For example, in the 1970s, it was reported that mast cells take up serotonin (5-hydroxytryptamine; 5-HT) into the cell [11]. In this study, we confirmed that noradrenaline-storing CTMCs express Oct3 (Fig. 5), which has been reported to take up histamine, serotonin, dopamine, and adrenaline into the cell, as well as noradrenaline [38]. All these bioactive substances have important physiological and pathophysiological functions, and as mentioned above, CTMCs may play a role in regulating their tissue concentrations. Similar to noradrenaline, adrenaline has been reported to promote cancer progression in a variety of cancer types. For example, adrenaline exposure has been reported to increase cancer stemness [39]. We have previously reported that adrenaline enhances cancer stemness in malignant peripheral nerve sheath tumors (MPNSTs) via β2-adrenergic receptor stimulation, thereby promoting cancer progression [40]. To further explore the role of mast cells in cancer biology, it would be meaningful to investigate whether noradrenaline-storing mast cells can store and release adrenaline.

Finally, we report the findings of this study, suggesting a possible role for mast cells. Although we understand that our study is limited by the lack of physiological studies that explain the role and importance of noradrenaline-storing CTMCs, we believe that sharing the discovery of these cells will benefit further research into the varied function and potential key roles of CTMCs. As detailed above, future physiological and pathophysiological experiments should be performed using in vivo transplantation and loss-of-function methods, such as knockdown of Oct3 genes in CTMCs.

## Conclusions

We found that a population of cells in several organs was positive for noradrenaline immunostaining. Further histological analyses using transgenic mice and in vitro studies revealed that connective tissue mast cells, a phenotypically distinct subpopulation of mast cells in rodents, take up, store, and release noradrenaline. We believe that these properties of mast cells are expected to be useful for addressing the gap in knowledge regarding the physiological dynamics of noradrenaline tissue distribution and regulation.

### Supplementary Information

**Additional file 1.** Representative images of negative controls of immunofluorescent analyses, related to Figure 1 and 5.

## Data Availability

The data that support the findings of this study are available from the corresponding author upon reasonable request.
