# Supplementary material for: Connective tissue mast cells store and release noradrenaline
Source: J Physiol Sci. 2023 Oct 12;73:24. doi: 10.1186/s12576-023-00883-3 (PMC10717848; doi:10.1186/s12576-023-00883-3)

Supplementary figure

Representative images of negative controls of immunofluorescent analyses, related to Figure 1 and 5.

(A and B) Representative images of sections from wild type mouse skin (A) and mammary gland (B) stained with anti-rabbit Alexa488 secondary antibody (without primary antibody), related to Figure 1A and 1B, respectively. Three independent experiments were performed.

(C and D) Representative images of sections from wild type mouse skin (C) and mammary gland (D) stained with anti-rabbit Alexa488, anti-goat Alexa594 and anti-rat Alexa647 secondary antibodies (without primary antibody), related to Figure 5C and 5D, respectively. Three independent experiments were performed.

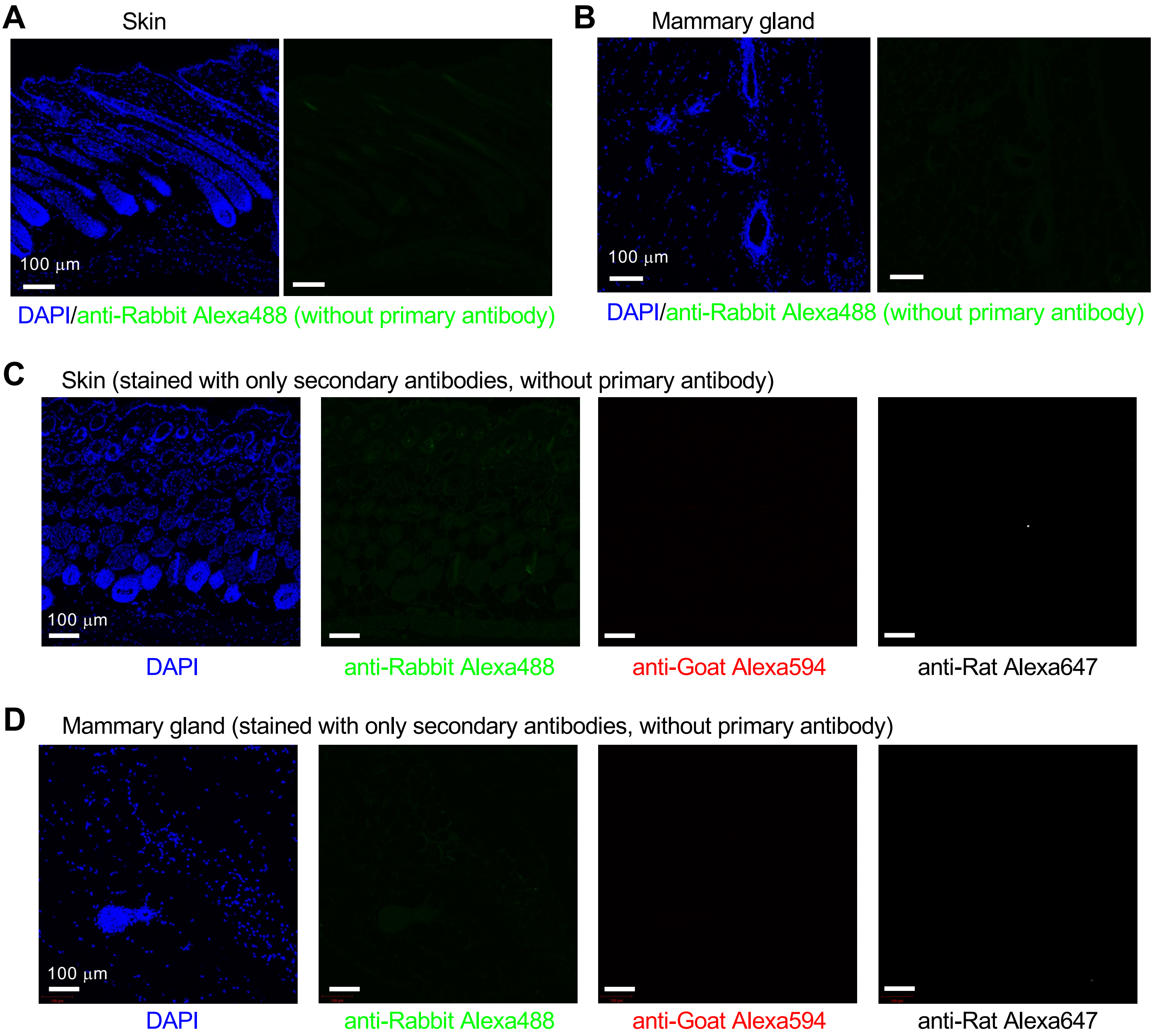

Supplement: Supplementary file 1 — Additional file 1. Representative images of negative controls of immunofluorescent analyses, related to Figure 1 and 5. [file 12576_2023_883_MOESM1_ESM.pdf]
